# Factors Related to Postoperative Pain Trajectories following Total Knee Arthroplasty: A Longitudinal Study of Patients Admitted to a Russian Orthopaedic Clinic

**DOI:** 10.1155/2016/3710312

**Published:** 2016-01-14

**Authors:** Nikolai Kornilov, Maren Falch Lindberg, Caryl Gay, Alexander Saraev, Taras Kuliaba, Leiv Arne Rosseland, Konstantin Muniz, Anners Lerdal

**Affiliations:** ^1^Department of Knee Surgery N 17, Russian Research Institute of Traumatology and Orthopaedics n.a. R.R. Vreden, Saint Petersburg 195427, Russia; ^2^Department of Surgery, Lovisenberg Diakonale Hospital, 0440 Oslo, Norway; ^3^Department of Nursing Science, Institute of Health and Society, Faculty of Medicine, University of Oslo, 0318 Oslo, Norway; ^4^Department of Family Health Care Nursing, University of California, San Francisco, CA 94143-0606, USA; ^5^Department of Research and Development, Lovisenberg Diakonale Hospital, 0440 Oslo, Norway; ^6^Department of Knee Surgery N 10, Russian Research Institute of Traumatology and Orthopaedics n.a. R.R. Vreden, Saint Petersburg 195427, Russia; ^7^Department of Research and Development, Division of Emergencies and Critical Care, Oslo University Hospital, 0424 Oslo, Norway; ^8^Institute of Clinical Medicine, University of Oslo, 0316 Oslo, Norway

## Abstract

This study explores sociodemographic, clinical, and surgical factors in relation to pain trajectories during the first 3 days following total knee arthroplasty (TKA). 100 patients (mean age 63.5 ± 7.8 years and 93% female) consecutively admitted for uncomplicated primary TKA were prospectively included. Postoperative pain was assessed using pain diaries. Measures of preoperative pain, symptoms, daily functioning, quality of life, comorbidities, knee function, perioperative characteristics, and physical/biochemical parameters were also evaluated. All pain ratings decreased in the three days following surgery (*p* < .001) as well as the reported number of daily hours in moderate/severe pain (*p* < .001). Women reported more pain than men (*p* = .009). Pain trajectories did not differ by education, employment, cohabitation, or any patient clinical and biochemical characteristics but were significantly related to preoperative anxiety (*p* = .029). Patients reporting moderate/severe pain prior to surgery also reported more hours in moderate/severe pain on days 0–3 postoperatively (*p* = .029). Patients with surgeries longer than 90 min reported more hours of moderate/severe pain compared with patients who had shorter surgeries (*p* = .008), and similar results were observed for ratings of pain with activity (*p* = .012). In this sample, only female gender, higher levels of preoperative pain and anxiety, and longer surgical duration were associated with increased pain after TKA.

## 1. Introduction

Total knee arthroplasty (TKA) is known to be a very painful orthopedic procedure [1]. Despite the fact that individual surgical technique and corresponding amount of tissue damage are usually similar from case to case in uncomplicated primary TKA, the level of postoperative pain varies widely among patients [2]. About half of patients experience moderate or severe pain in the first days after surgery, and this pain may become even worse once rehabilitation is started [3, 4]. Effective pain control is therefore important for optimizing the rehabilitation process in order to achieve patient satisfaction with a good functional outcome as well as reduce hospitalization length and costs [5].

Surgeons try to reduce the tissue damage by shortening the length of skin, capsule, and extensor apparatus incisions, modifying the type of arthrotomy (e.g., quadriceps-sparing, minimidvastus, or subvastus approaches), reducing the amount of unnecessary soft-tissue releases, or optimizing tourniquet pressure and time [6]. In addition, various multimodal analgesic options have been developed and their different combinations are currently used in clinical practice [7, 8]: paracetamol, nonsteroidal anti-inflammatory drugs (NSAIDs), opioids, ketamine [9], alpha-2 adrenergic agonists [10], corticosteroids [11], gabapentinoids, local infiltration analgesia (LIA), prolonged epidural anesthesia, continuous or single-shot peripheral nerve blocks, and cryotherapy.

All of these options have established advantages and disadvantages both from clinical and economical points of view [12, 13], but consensus on what protocols are most beneficial among different patient subgroups is still lacking [14]. Despite the fact that modern multimodal approaches to analgesia have decreased the prevalence of persistent postsurgical pain after TKA, further improvement is necessary.

There is growing attention to cognitive and emotional patient characteristics that together with other multiple factors like age, gender, ethnicity, level of preoperative pain, and length of waiting time before the surgery may influence the amount of acute and chronic postoperative pain after knee replacement [15–17]. Negative psychological conditions like anxiety, depression, sleep disturbance, pain catastrophising, hypervigilance, and perceived injustice were found to be very important [18–22].

Thus, the aim of this study was to explore sociodemographic, clinical, and surgical factors in relation to pain trajectories during the first 3 postoperative days following total knee arthroplasty (TKA).

## 2. Materials and Methods

### 2.1. Patients and Study Procedures

This study is the first report from a prospective longitudinal study of pain, symptoms, and health-related quality of life in 100 consecutive Caucasian patients receiving total knee arthroplasty. The study was conducted at the Russian Research Institute of Traumatology and Orthopedics n.a. R.R. Vreden (Vreden's Institute) in St. Petersburg, Russia. The study was approved by the local Ethics Committee at Vreden's Institute. Informed written consent was obtained from all patients.

Patients admitted to Vreden's Institute for TKA were recruited for the study between April and September 2014. Patients were included if they were ≥18 years of age; were able to read, write, and understand Russian; and were scheduled for unilateral primary TKA. Patients were excluded if they underwent unicompartmental or revision surgery, had prior TKA on the contralateral knee, or had primary TKA complicated by one of the following conditions: extensive bone defects requiring substitution by graft or metal, collateral ligament insufficiency, frontal deformities over 30 degrees, hyperextension >15 degrees, fibrous or bony knee ankylosis, femur or tibia extra-articular deformities in more than one plane and over 10 degrees, extensor mechanism deficit, or hip fusion.

Patients received written information about the study on the day of admission. Patients were admitted to the hospital between 1 and 7 days prior to surgery depending on comorbidities. Patients who met the inclusion criteria were invited to participate by the senior surgeons (NK and TK) on the day of admission. After obtaining written informed consent, patients completed a questionnaire that assessed demographic characteristics, preoperative symptoms, and psychological factors. Preoperative pain was assessed the day before surgery, and acute postoperative pain was assessed using a 4-day pain diary, which was completed every evening starting on the day of surgery (Day 0) until postoperative Day 3. The completed questionnaires and the pain diary were collected by the junior surgeons (AS and KM).

### 2.2. Surgical, Anesthesiological, and Pain Management Procedures

The surgery, anesthesia, and postoperative pain management procedures were standardized. All patients received the primary cemented unconstrained posterior cruciate retaining or substituting implants for the TKA. A tourniquet was used during surgery in a minority of cases (21%). To avoid potential harm to the arterial or vein wall, tourniquet was not used in patients with comorbidities, such as varicose vein disease, atherosclerosis of peripheral arteries, diabetes, and other factors associated with increased risk of venous thromboembolism. Because the application of tourniquet within recommended pressure range is ineffective for patients with excessive subcutaneous fat in the middle of the thigh, tourniquet was also not used for obese patients. The low rate of tourniquet use was due to these contraindications. The less-invasive medial parapatellar approach was utilized in all patients without eversion or resurfacing of patella [23]. Drains were placed and removed on postoperative Day 1.

Neuraxial block with bupivacaine and sedation were used for anesthesia for all patients. Spinal anesthesia has been the method of choice for TKA patients in Vreden's Institute for the last two decades because it is safe, well tolerated by patients with comorbidities, and not only clinically effective, but cost-effective as well. In one patient, an incomplete effect of the spinal anesthesia was noticed, so general anesthesia was used as an adjunct because the patient refused a peripheral nerve blockade. Although prolonged epidural anesthesia was previously widely utilized in TKA patients during surgery and even for a few days after it, it is now rarely used at our institution for several reasons. First, the achieved block is more variable in strength and the failure rate of the technique may reach 28% [24]. Second, some patients experience muscle weakness that postpones early ambulation. Third, hypotension and urinary retention are additional concerns. The last but probably most important reason is that the use of direct anticoagulants in the postoperative period makes all our patients ineligible for this technique.

For postoperative pain management, all patients received an intramuscular (IM) injection of ketoprofen 100 mg 3 times per day until discharge from the clinic. If this did not provide adequate pain relief on the first day, the second line drug was a single IM injection of opiate trimeperidine (Promedol) (0.01 mg). On the second and third days, the second line drugs were tramadol (100 mg IM twice per day) or Zaldiar (1 pill every 6 hours). This has been the standard pain management at our institution for many years. All patients received a single intravenous injection of Dexamed (Dexamethasone) (8 mg) just before incision.

Mobilization and physical therapy were standardized. After surgery, patients spent several hours in the recovery room supervised by an anesthesiologist. Patients were then returned to the ward and remained in bed until the next morning when physical therapy was initiated. Beginning on postoperative Day 1, a physiotherapist mobilized the patients out of bed, assisted them with exercises, and helped them ambulate on crutches with partial weight bearing on the operated leg. During the following days, patients received daily physical therapy, which included ambulation as tolerated, and performed flexion and extension of the knee exercises two to three times daily. Most patients were discharged on postoperative Day 7.

### 2.3. Clinical and Perioperative Characteristics

Data on brand of implant, American Society of Anesthesiologists (ASA) physical status classification [25], length of surgery, tourniquet use, infections (i.e., deep prosthetic, wound), as well as comorbidities, preoperative blood pressure, hemoglobin, C-reactive protein, and creatinine levels were obtained from medical records. Data on anesthesia regimen and postoperative pain medications were obtained from patients' medical records.

### 2.4. Preoperative Pain

*The Brief Pain Inventory (BPI).* Patients' level of preoperative pain and its impact on function were assessed using the BPI [26]. The BPI consists of four items that measure pain intensity on a 0 to 10 numeric rating scale (NRS); one item that measures pain relief; a body map to assess pain locations; and seven items that measure interference with function. The Russian version of the BPI has well-established psychometric validity and reliability [27]. For this analysis, the level of average pain reported preoperatively was used to categorize those with moderate or severe pain (NRS ≥ 4) or severe pain (NRS ≥ 7).

### 2.5. Acute Postoperative Pain

*Pain Diary.* Patients rated their acute postoperative pain every evening starting on the day of surgery and continuing through Day 3. Six different aspects of pain were rated (i.e., worst, average, least, with activity, at rest, and duration), but this analysis emphasizes pain with activity and pain duration given their potential to impact physical rehabilitation [28]. Patients rated their pain using a 0 (no pain) to 10 (worst imaginable pain) NRS and estimated their pain duration as the number of hours in the prior 24 hours with moderate to severe pain (i.e., rated on the NRS as 4 or higher).

### 2.6. Symptom Measures

Several measures were used to assess patients' other symptoms. The 14-item Hospital Anxiety and Depression Scale (HADS) [29] was used to evaluate anxiety and depression in the past week; a score above 8 on either the anxiety or depression subscale is clinically significant. The Pittsburgh Sleep Quality Index was used to assess sleep disturbance in the past month, and scores above 5 are considered indicative of poor sleep. The 7-item Fatigue Severity Scale (FSS-7) [30] was used to evaluate fatigue interference during the past week. Total scores can range from 1 to 7 with higher scores indicating higher levels of interference. The FSS-7 has good psychometric properties among adults with a variety of medical conditions. For this study, the FSS was translated into Russian and its Cronbach's alpha was 0.93.

### 2.7. Functional Ability and Quality of Life Measures

The Knee Society Score (KSS) [31] and Function Score were used to assess the level of knee function. The Knee Score is based on 9 items, of which 3 assess patient-reported pain and 6 involve physiotherapist assessments of range of motion, stability, and alignment. The Function Score consists of 5 items assessing the patient's ability to walk (i.e., maximum distance and up and down stairs) and rise from a chair, as well as their use of support devices (i.e., cane, crutches, and walker). Both scores range 0–100, with higher scores indicating better knee function.

The Lawton Instrumental Activities of Daily Living (IADL) Scale [32] was used to assess patients' abilities to perform everyday tasks. The scale consists of 8 items assessing domains such as shopping, housekeeping, transportation, and finances. Mean substitution was used for up to two items the patient did not consider applicable to them, typically because someone else in the household managed that task. Total scores were considered missing when more than 2 items were not answered or were not considered applicable. Scores range 0–8, with higher scores indicating greater independence with activities of daily living.

The EQ-5D-3L [33] was used to assess patients' quality of life. The scale consists of 5 items assessing the following 5 domains: mobility, self-care, usual activities, pain/discomfort, and anxiety/depression. Each item is rated on a 3-point scale: no problems, some problems, extreme problems. In addition, a visual analogue scale (VAS) is used to assess the patient's self-rated health on a 0–100 scale, where 0 indicates “worst imaginable health state” and 100 indicates “best imaginable health state” [34]. Only the EQ VAS was used in this analysis, and scores of 80 and higher were considered indicative of good health-related quality of life.

### 2.8. Statistical Analyses

Descriptive statistics and frequency distributions were performed on the preoperative demographic and clinical sample characteristics. Independent sample *t*-tests and chi-squared tests were used to compare groups on continuous and categorical variables, respectively. Fisher's Exact Test was used instead of the chi-square test when expected cell frequencies were below 5. Repeated measures analysis of variance was used to evaluate the within-subjects effect of time evaluated at 4 points (i.e., Days 0–3) and various between-subjects effects (i.e., sociodemographic, clinical, and surgical factors).  Table 1 presents a list of the proposed predictors that was developed based on a review of the literature on perioperative pain intensity in patients undergoing TKA [35–39]. For continuous measures without established cut-points, a median split was used to compare the pain trajectories of patients scoring relatively high and low. All analyses were conducted using SPSS version 22 (IBM, Armonk, NY). A *p* value of <.05 indicated statistical significance.

## 3. Results

### 3.1. Sample Characteristics

Of the 241 knee arthroplasty patients evaluated for inclusion in the study, 14 were not eligible because they had had prior knee replacement, 12 were transferred to other departments due to the necessity of other surgeries prior to the TKA, 23 were discharged prior to surgery due to severe comorbidities, and 92 declined to participate. The remaining 100 patients were enrolled in the study and are included in the analysis. Demographic, clinical, and surgical characteristics are summarized in Table 1. The sample was mostly female (93%) and had a mean age of 63.5 (±7.8) years.

### 3.2. Pain Trajectories

**Figure 1:**
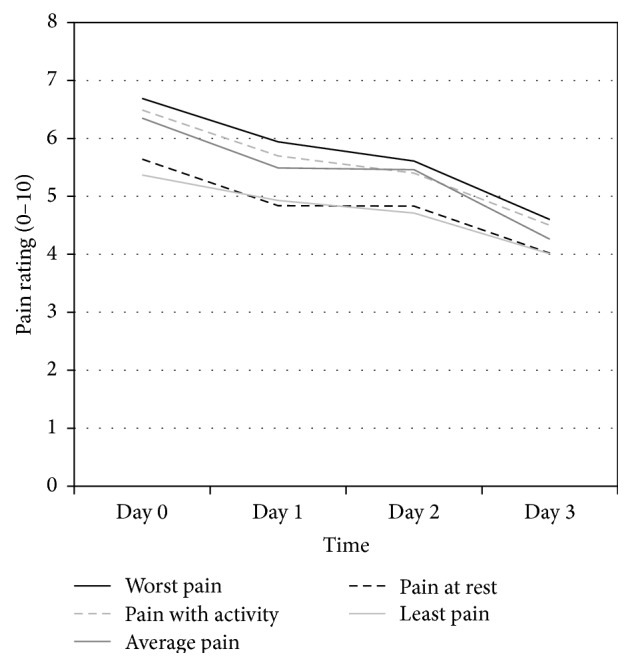
Pain severity ratings from day of knee replacement surgery (Day 0) through the third postoperative day. The five types of pain ratings all decreased in a similar pattern across the first three days after surgery (all main effects for time had *p* < .001). Hours in moderate to severe pain (NRS ≥ 4) are not included in the figure but showed a similar decreasing trend over time (*p* < .001).

As shown in Table 2 and Figure 1, all pain ratings decreased in the three days following knee surgery (all main effects for time, *p* < .001). Similarly, the reported number of hours per day in moderate/severe pain (i.e., NRS ≥ 4) decreased over time (*p* < .001). Given the similar trajectories for the different pain ratings, subsequent analyses focus only on pain with activity as a measure of pain intensity and hours in moderate to severe pain as a measure of pain duration or burden.

### 3.3. Pain by Sociodemographic Characteristics

Women reported more pain than men across Days 0–3 (average pain ratings of 5.5 (SD 1.62) for women versus 3.8 (SD 1.34) for men, *p* = .009), but with only 7 men in the sample, this may not be a robust finding. Age had modest negative correlations (range of −0.20 to −0.25) with pain ratings on Days 1 and 2 but was not correlated with pain ratings on Day 0 or Day 3. Pain trajectories did not differ by education, employment, or cohabitation status.

### 3.4. Pain by Clinical Characteristics

**Figure 2:**
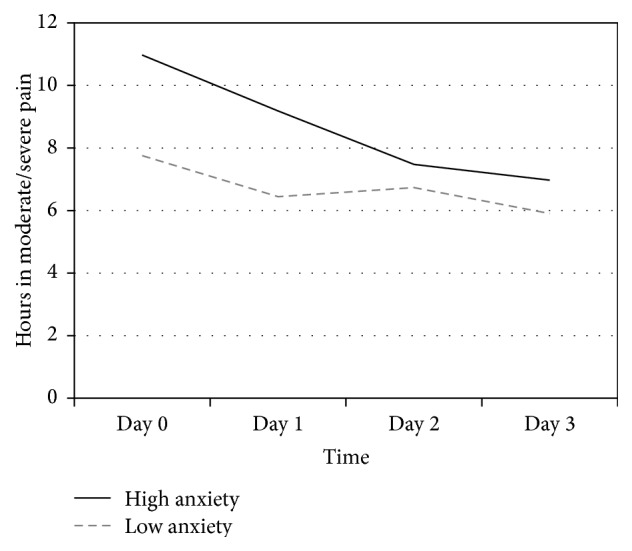
Hours of moderate to severe pain over time by preoperative anxiety. Patients with more preoperative anxiety (HADS anxiety score >8) reported more hours per day in moderate to severe pain compared with patients who had less anxiety (HADS score ≤8; main effect for anxiety, *p* = .029; interaction with time, *p* = .046).

Pain trajectories from the day of surgery through the first three postoperative days were largely unrelated to patient clinical characteristics. Pain ratings over time did not differ based on the patient's preoperative Knee Society Score, Function Score, their level of independence with activities of daily living, or their health-related quality of life. In addition, pain ratings did not differ based on patient ratings of preoperative sleep quality (PSQI), fatigue (FSS-7), or depression (HADS). However, pain ratings over time were significantly related to preoperative anxiety, with the patients who reported higher anxiety reporting more pain with activity, but only on the day of surgery, not on the subsequent three postoperative days (interaction of time and anxiety *p* = .034). As shown in Figure 2, anxious patients also reported more hours in moderate/severe pain, particularly on the day of surgery and first postoperative day (main effect for anxiety *p* = .029; interaction between anxiety and time, *p* = .046). Not surprisingly, patients who reported moderate to severe average pain* prior to surgery* also reported more hours in moderate to severe pain on Days 0–3* postoperatively* (*p* = .029). However, preoperative average pain rating was unrelated to ratings of pain with activity on postoperative Days 0–3.

### 3.5. Pain Management

All patients received spinal anesthesia and sedation, except one who received total intravenous anesthesia (TIVA). Excluding the one patient with TIVA did not alter any of the results. Medications administered to patients to manage postsurgical pain are summarized in Table 3. There was little variation in the medications administered on Days 1–3, and analysis of medication regimen on the day of surgery (Day 0) indicated no relationship to pain ratings. Dose information was not available for this analysis.

### 3.6. Pain by Surgical Characteristics

#### 3.6.1. Surgery Duration

**Figure 3:**
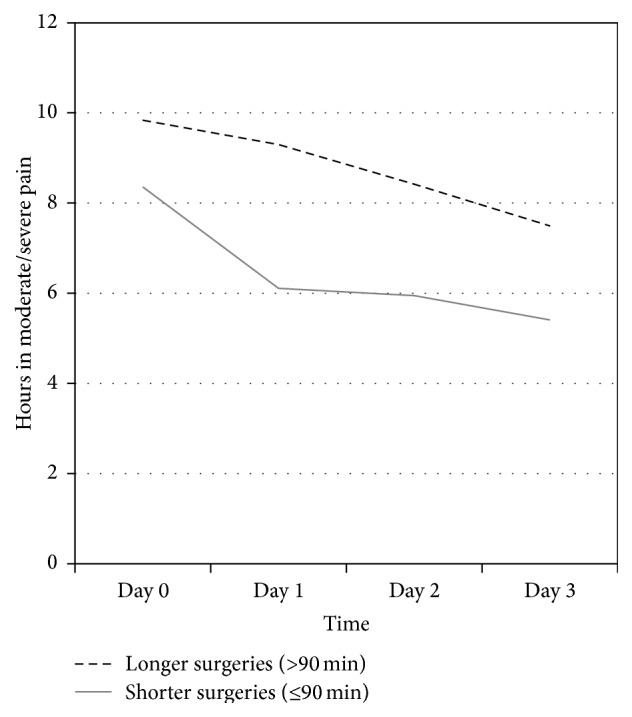
Hours in moderate/severe postoperative pain by duration of surgery. Longer knee replacement surgeries were associated with consistently more hours in moderate/severe postoperative pain (NRS ≥ 4) compared with shorter surgeries (*p* = .008). Similar difference was observed for ratings of pain with activity in relation to surgery duration (*p* = .012).

The mean surgery duration was 95.6 (SD 23.6) minutes, with a median of 90 minutes. A comparison of shorter (<90 minutes) and longer (>90 minutes) surgeries indicated that longer surgeries were associated with higher postoperative pain ratings. As shown in Figure 3, patients with longer surgeries reported consistently more hours per day of moderate/severe pain (NRS ≥ 4) compared with patients who had shorter surgeries (*p* = .008). Similar results were observed for ratings of pain with activity (*p* = .012).

#### 3.6.2. Implant Type

**Figure 4:**
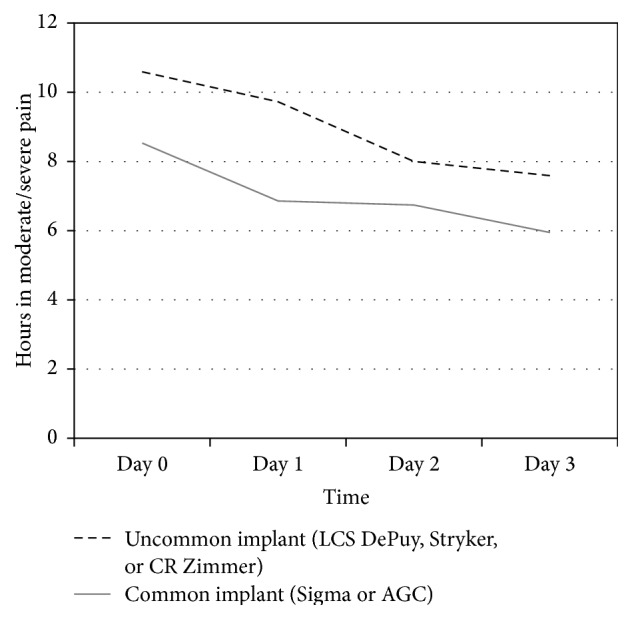
Hours of moderate to severe pain by implant type. The more commonly used implants were associated with slightly fewer hours per day in moderate to severe postoperative pain than the less commonly used implants, although the difference did not reach statistical significance (*p* = .060).

Five different types of knee replacement implants were used in this study, with varying frequencies of use. The Scorpio NRG (Stryker, Kalamazoo, MI, USA) and NexGen (Zimmer Biomet, Warsaw, IN, USA) were only used 4 or 5 times each and were grouped with the LCS DePuy (Johnson & Johnson, Warsaw, IN, USA) for the purpose of analysis. The Sigma DePuy (Johnson & Johnson, Warsaw, IN, USA) and AGC (Zimmer Biomet, Warsaw, IN, USA) were the most commonly used implants and were utilized in 77% of the surgeries in this study. Although the difference did not reach statistical significance (*p* = .060), the less commonly used implants (i.e., LCS, Scorpio NRG, and NexGen) were associated with slightly more hours in moderate/severe pain on Days 0–3 than the more commonly used implants (i.e., the Sigma and AGC) (Figure 4). The implants did not differ with respect to postoperative pain with activity.

### 3.7. Factors Related to Surgery Duration

As shown in Tables 4 and 5, several factors were related to longer surgery duration, particularly implant type, amount of bleeding, preoperative hemoglobin value, and days in hospital. The more commonly used implants (i.e., Sigma and AGC) were associated with shorter surgery durations compared to the less commonly used implants (i.e., LCS, Scorpio NRG, and NexGen). One of the patients who received the NexGen implant had a surgery duration of 210 minutes, but a sensitivity analysis excluding this patient (*p* = .004) and the nonparametric Kruskal-Wallis test (*p* < .001) confirmed robust differences in surgery duration by implant type. Longer surgeries were also associated with more bleeding, but surgery duration was unrelated to tourniquet use. Longer surgeries were also associated with lower preoperative hemoglobin values, but surgery duration was unrelated to postoperative hemoglobin values. Surgery duration was also unrelated to creatinine and CRP values. Although surgeries longer than 90 minutes were not significantly associated with longer preoperative or postoperative stays in hospital, they were associated with longer hospital stays overall (i.e., combining pre- and postoperative days in hospital).

## 4. Discussion

A primary finding of this study was that the pain management protocol implemented at our institution was providing insufficient pain relief for some patients. Patients reported mean levels of pain in the moderate to severe range (NRS ≥ 4) on the day of surgery and first three postoperative days. Patients also reported a mean of 9 hours per day in moderate to severe pain. As a result of these findings, new modalities, such as local infiltration analgesia during surgery and intravenous Perfalgan (paracetamol) (Agen, France), were recently added to the protocol. In addition, single-injection femoral nerve blocks are now also used more widely. The levels of pain reported by the patients in this study may have also impacted the study results. The factors evaluated may have somewhat subtle effects that might be more evident among patients with greater pain relief. Thus, due to the possibility of ceiling effects, negative findings should be interpreted with caution. Nonetheless, our study identified several factors as predictors of heightened pain experience, even in this more limited pain management context.

Our study evaluated a variety of different psychological and clinical preoperative patient characteristics and found that only female gender and higher levels of preoperative anxiety and pain were associated with higher levels of pain reported in the early postoperative period after TKA. Anxiety is a recognized risk factor for persistent postsurgical pain [19]. Roth et al. [39] have found that high catastrophisation is predictive of greater postoperative pain, specifically pain at Day 2 and later following TKA. Ip et al. [40] mentioned both presurgical anxiety and psychological distress as predictive factors of postoperative pain intensity. Pinto et al. [41] suggested that pain catastrophising may act as a mediator in the relationship between preoperative anxiety and acute postsurgical pain. Preoperative anxiety is an important pain predictor not only for the first days after surgery but also at 6 weeks [42] and 6 months [43] and has even been linked to residual long-term pain after TKA [16, 44].

Numerous publications indicate that patients who are depressed preoperatively have worse results after TKA surgery in terms of pain, functionality, and satisfaction [17, 45, 46]. In our cohort of patients, we did not find any association between depression and pain trajectories. This is consistent with a recent study by Riddle et al. [47] in which they found no association between preoperative depressive symptom severity and postoperative pain. Furthermore, preoperative and postoperative depressive symptoms in patients before and after TKA did not appreciably change over a 6-year perioperative period. Pérez-Prieto et al. [48] also showed that although depressed patients have worse outcomes after TKA surgery, they also tend to start with worse pain and function levels preoperatively and the degree of improvement is the same as that for nondepressed patients. They conclude that it is equally worthwhile for depressed patients to undergo TKA surgery as is the case for nondepressed patients because to some extent the surgery may also cure the depression.

Other reported predictors for increased postoperative pain include female gender, younger age, increased BMI, increased severity of preoperative pain at the surgical site, preoperative use of opioids, general anesthesia, preoperative use of anticonvulsants, and preoperative use of antidepressants [36]. Among our cohort of patients, we observed similar associations with gender and preoperative pain but found no association with age and could not evaluate the remaining factors. Women reported more pain than men (*p* = .009), but the male subgroup consisted of only 7 patients, which reflects the typical gender distribution among primary TKA patients in our institution in recent years [49]. Patients who had a moderate to severe level of preoperative pain also reported more hours of moderate to severe pain during the first 3 postoperative days, (*p* = .029), although preoperative pain was unrelated to postoperative pain in activity. Neuropathic pain was not addressed in the current study, but Lavand'homme et al. [35] found that, during the first week after TKA, a subgroup of patients whose pain had a neuropathic component reported worse maximal pain and pain associated with mobilization, while no differences were demonstrated in trajectories of pain at rest.

The second finding of the study was that, among intraoperative variables, surgeries longer than 90 minutes were associated with more postoperative pain in activity, as well as more hours of moderate to severe pain. This finding is consistent with those of Niki et al. [50] that reduction of the duration of the surgical procedure as well as minimization of tissue damage and decrease of tourniquet time may also play a role in postoperative recovery [50]. Unexpectedly, surgery time was related to the brand name of the implant. The most commonly used among this cohort of patient implants, Sigma and AGC (77% of all cases), were associated with shorter surgery duration and correspondingly less postoperative pain than the other less commonly used brands. The implant choice was performed randomly the day before surgery, depending upon availability of instrument sets, taking into consideration that the indications for using the primary TKA implants do not differ between the manufacturers. All surgeons involved in the study have vast experience in knee arthroplasty with over 500 implantations per year in total. All of the less commonly used implants in the study were familiar to the surgeons for several years, so it is unlikely that this finding is explained by a learning curve with these instrumentation systems. While this finding may be due to the fact that the more commonly a specific instrument is used, the faster the surgery is performed, it could also reflect the relative ease of implementation for the different systems.

This study has several strengths and limitations that need to be considered. A strength of the study is that Vreden's Institute used a standard protocol for patient care, thereby limiting the number of potential variables that may have influenced the results. In addition, the study evaluated a fairly comprehensive set of factors, including demographics, psychological well-being, knee function, physiological parameters, and intrasurgical factors, as well as an array of pain measures, including a 4-day pain diary. However, the sample size was small and likely underpowered for the number of factors being evaluated, and the sample was relatively homogeneous, particularly with respect to gender. Although the sample's gender distribution is representative of the patient population admitted for TKA at Vreden's Institute, applying the study findings to male patients should be done with caution, given the small number of men included in the study and the observed gender differences in pain. The surgeries were performed by several different surgeons, which may have added some individuality in soft-tissue handling. Nevertheless, all were trained by senior surgeon (NK) to do the surgery methodically and in a manner corresponding to the principles of less invasive surgery, such as dissecting as little tissue as possible, minimizing the length of incision during approach, avoiding excessive tension by retractors, utilizing the principle of mobile window during all steps of TKA, avoiding eversion of the patella or full subluxation of the tibial plateau anteriorly, and keeping soft-tissue releases minimal but sufficient for correction of both alignment and contracture, as well as gap balancing. Finally, although the pain management protocol was standardized, with little variation in dosing, this study did not include an extensive evaluation of the patients' pain management, which seemed to provide inadequate pain relief for some patients. Future research is needed to evaluate the complex relationships between the timing and dosing of pain management medication and postoperative pain trajectories.

## 5. Conclusions

Postoperative pain levels were generally high in this sample of TKA patients. Of the numerous previously reported medical and psychological comorbidities associated with pain following TKA, only gender, anxiety, and a higher level of preoperative pain were associated with increased postoperative pain after TKA in our cohort of patients. Among the surgical factors examined, surgery duration, which varied by the brand of implant, was associated with postoperative pain trajectories. Better understanding of which TKA patients are at greatest risk for postoperative pain will allow health care providers to individualize both preventive and protective analgesic peri- and postoperative treatments [35, 51].

## Figures and Tables

**Table 1:** Sample Characteristics and their Relationship to Pain on Postoperative Days 0 to 3.

	Mean (SD)	% (*n*)	Relationships to pain severity in activity (*p* < .10)	Relationships to hours in moderate to severe pain (*p* < .10)
*Demographic characteristics*				
Age in years, (range 47–81)	63.5 (7.8)		None (using cutpoint of 65)	None (using cutpoint of 65)
Sex			Main effect for sex, *p* = .052 (more pain for females)	Interaction of sex and time, **p** = .032 (more hours of pain for females)
Male		7% (7)		
Female		93% (93)		
Living situation			None	None
Lives alone		18% (18)		
Lives with spouse		58% (58)		
Lives with other(s)		24% (24)		
Education			None	None
Less than high school		45% (45)		
Completed high school		55% (55)		
Employment status			None	None
Paid work or self-employed		24% (24)		
No paid work or retired		76% (76)		

*Preoperative clinical characteristics*				
EQ VAS health-related quality of life (0–100 scale)	55.6 (15.6)		Main effect for EQ VAS, *p* = .061 (healthier patients reported slightly more pain)	None
<80		88% (88)		
Fatigue Severity Scale (1–7 scale)	3.17 (1.66)		None (using cutpoint of either 4 or 5)	None (using cutpoint of either 4 or 5)
≥5		14% (14)		
≥4		28% (28)		
Pittsburgh Sleep Quality Index	8.35 (3.50)		None (using cutpoint of 5)	None (using cutpoint of 5)
>5		73% (73)		
HADS Depression Subscale	6.12 (4.03)		None	None
0–8 (none)		70% (70)		
9-10 (mild)		17% (17)		
>10 (moderate/severe)		13% (13)		
HADS Anxiety Subscale	7.34 (4.18)		Interaction of anxiety and time, **p** = .034 (anxious patients reported more pain on day 0, but not after	Main effect of anxiety, **p** = .029; interaction of anxiety and time, **p** = .046 (anxious patients had more hours of pain, particularly on days 0 and 1)
0–8 (none)		61% (61)		
9-10 (mild)		20% (20)		
>10 (moderate/severe)		19% (19)		
BPI Average preoperative pain rating (0–10 scale)	4.76 (2.54)		None (using cutpoint of either 4 or 7)	Main effect for pre-operative pain ≥4, **p** = .029 (patients with more pre-op pain reported more hours of post-op pain)
≥4 (moderate or severe pain)		67% (67)		
≥7 (severe pain)		24% (24)		
IADL (0–8 scale) (*n* = 95)^a^	6.43 (1.10)		None (median split at 6)	None (median split at 6)
KSS Knee Score (0–100 scale)	33.5 (20.3)		None (median split at 30)	None (median split at 30)
KSS Function score (0–100 scale)	45.2 (17.1)		None (median split at 44)	None (median split at 44)

*Surgery characteristics*				
Anesthesia			Not analyzed	Not analyzed
Spinal/sedation		99% (99)		
TIVA		1% (1)		
Surgery duration in minutes (range 50–210)	95.6 (23.6)		Main effect for surgery duration, **p** = .012 (more pain for surgeries >90 minutes)	Main effect for surgery duration, **p** = .008 (more hours of pain for surgeries >90 mins)
Implant type			None	Main effect for implant, *p* = .060 (common implants [first 2] had slightly less hours of pain than uncommon implants [last 3])
Sigma (DePuy Johnson & Johnson)		43% (43)		
AGC (Zimmer Biomet)		34% (34)		
LCS (DePuy Johnson & Johnson)		14% (14)		
Scorpio NRG (Stryker)		5% (5)		
NexGen (Zimmer Biomet)		4% (4)		
Tourniquet use		21% (21)	None	None
Bleeding (ml)	341.7 (183.2)		None	Main effect for bleeding, *p* = .085 (slightly more hours of pain for bleeding > 300 ml)
Hemoglobin (g/L)			Main effect for preop Hgb, *p* = .086 (slightly more pain among patients with preop Hgb ≥ 120). When only women are included, **p** = .039. No effects for postop Hgb, but the numbers with normal postop Hgb were small.	Main effect for preop Hgb, *p* = .071 (slightly more hours of pain among patients with higher preop Hgb ≥ 120). When only women are included, **p** = .045. No effects for postop Hgb, but the numbers with normal postop Hgb were small.
Females (*n* = 93)				
Pre-operative (% (*n*) < 120)	128.6 (12.0)	26% (24)		
Day 0 at 20:00 (% (*n*) < 120)	106.9 (11.7)	89% (83)		
Day 4 (% (*n*) < 120)	99.0 (13.4)	92% (86)		
Males (*n* = 7)				
Pre-operative (% (*n*) < 120)	139.1 (12.3)	0% (0)		
Day 0 at 20:00 (% (*n*) < 120)	110.1 (11.9)	71% (5)		
Day 4 (% (*n*) < 120)	106.4 (14.4)	86% (6)		
C-reactive protein, preoperative (mg/L)	4.07 (5.40)		None (split at 5)	None (split at 5)
≥5		24% (24)		
Creatinine, preoperative (mcmol/L)			None (split at 44)	None (split at 44)
Female (*n* = 93) (% (*n*) < 44)	63.9 (16.5)	5% (5)		
Males (*n* = 7) (% (*n*) < 44)	79.8 (15.7)	0% (0)		
Length of hospital stay in days			None (median split at 16)	None (median split at 16)
Pre-operative	7.2 (4.1)			
Post-operative	9.2 (2.4)			
Total	16.4 (4.3)			

Note. Relationships to pain were determined by including each variable as a between-subjects factor in a repeated measures analysis of pain in activity and hours in moderate/severe pain. Variables with gender differences were evaluated both with and without men.

^a^Five patients were missing IADL total score because they answered “does not apply” to more than 2 of the IADL items.

**Table 2:** Pain ratings on Days 0–3 after knee replacement surgery.

	Mean pain rating (SD)			
	Day 0	Day 1	Day 2	Day 3
Average pain	6.35 (2.35)	5.49 (2.11)	5.46 (2.20)	4.26 (1.86)
Least pain	5.37 (2.44)	4.93 (2.24)	4.71 (2.36)	4.01 (2.24)
Worst pain	6.69 (2.46)	5.94 (2.40)	5.61 (2.29)	4.60 (1.98)
Pain at rest	5.64 (2.20)	4.84 (2.20)	4.83 (2.09)	4.02 (2.14)
Pain with activity	6.49 (2.41)	5.70 (2.41)	5.40 (2.35)	4.50 (2.16)
Hours > NRS4	8.99 (6.69)	7.46 (5.22)	7.02 (4.75)	6.31 (4.54)

Effects of change over time, all *p* < .001.

**Table 3:** Pain management 0–96 hours after knee replacement surgery.

	0–24 hours	24–48 hours	48–72 hours	72–96 hours
Trimeperidine (Promedol) (Moscow, Russia), %	30%	—	1%	—
Ketoprofen (ketoprofen) (Stavropol, Russia), %	81%	98%	98%	97%
Tramadol (tramadol) (Novokuznetsk, Russia), %	56%	7%	—	—
Paracetamol and tramadol (Zaldiar) (Aachen, Germany), %	3%	7%	5%	5%

*Note.* Each percentage refers to the proportion of patients who received the listed medication during the indicated time period.

**Table 4:** Descriptive surgical and clinical characteristics by duration of surgery.

Characteristic	Surgery duration		Statistics
	≤90 minutes	>90 minutes	
Implant type, % (*n*)			All 5, Fisher's exact **p** < .001
Sigma	47.4% (27)	37.2% (16)	Sigma versus AGC versus others
AGC	43.9% (25)	20.9% (9)	*χ* ^2^(2) = 16.0, **p** < .001
Other implants:	21.7% (5)	78.3% (18)	Common versus uncommon
*LCS*	35.7% (5)	64.3% (9)	*χ* ^2^(1) = 15.2, **p** < .001
*Scorpio NRG *	0% (0)	100% (5)	
*NexGen *	0% (0)	100% (4)	
Tourniquet use, % (*n*)	24.6% (14)	16.3% (7)	*χ* ^2^(1) = 1.01, *p* = .314
Bleeding, mL, mean (SD)	297 (142)	401 (215)	*t*(98) = 2.93, **p** = .004
Hemoglobin, mL			
Preoperative, mean (SD)	126.9 (11.4)	132.6 (12.9)	*t*(98) = 2.34, **p** = .022
Day 0 at 20:00, mean (SD)	106.3 (11.9)	108.1 (11.4)	*t*(98) = 0.78, *p* = .437
Day 4, mean (SD)	100.6 (13.7)	98.2 (13.3)	*t*(98) = 0.88, *p* = .379
Creatinine, Day 0	63.2 (13.3)	67.3 (20.5)	*t*(98) = 1.20, *p* = .231
C-reactive Protein, Day 0	4.23 (4.68)	3.86 (6.29)	*t*(98) = 0.33, *p* = .742
Days in hospital, mean (SD)			
Preoperative	6.7 (4.4)	8.0 (3.6)	*t*(98) = 1.53, *p* = .128
Postoperative	8.9 (2.0)	9.5 (2.9)	*t*(98) = 1.15, *p* = .255
Total	15.6 (4.1)	17.4 (4.4)	*t*(98) = 2.14, **p** = .035

*Note.* Surgery duration did not differ by patient age or sex. Common implants were Sigma and AGC, and uncommon implants were LCS, Scorpio NRG, and NexGen.

**Table 5:** Descriptive characteristics of surgery duration (minutes), tourniquet use, and postsurgical hemoglobin for five different types of knee replacement implants.

	*n*	Surgery duration^a^ Mean (SD)	Tourniquet use^b^ % (*n*)	Bleeding, mL Mean (SD)	Hemoglobin, g/L^c^		Postoperative length of stay Mean (SD)
					Day 0, 20:00 Mean (SD)	Day 4 Mean (SD)	
Sigma	43	93.6 (20.0)	11.6% (5)	381 (172)	107.9 (11.7)	101.4 (15.0)	9.60 (2.89)
AGC	34	87.8 (20.6)	26.5% (9)	291 (165)	108.4 (10.9)	101.0 (12.1)	8.74 (1.68)
Other implants:	23	110.7 (27.7)	30.4% (7)	343 (215)	103.7 (12.5)	93.8 (11.5)	8.91 (2.21)
*LCS*	*14*	*105.4 (21.1)*	*50.0% (7)*	*318 (192)*	*100.4 (9.2)*	*91.1 (11.5)*	*8.29 (2.16)*
*Scorpio NRG*	*5*	*104.0 (5.5)*	*0%*	*470 (311)*	*110.0 (4.9)*	*99.4 (8.5)*	*10.80 (1.64)*
*NexGen*	*4*	*137.5 (49.9)* ^a^	*0%*	*275 (126)*	*107.0 (24.8)*	*96.5 (14.2)*	*8.75 (2.06)*

*Note.* Italicized text represents the 3 types of implants grouped together as “other implants.” Implant types did not differ with respect to bleeding, length of postoperative hospital stay, creatinine values (not shown), or CRP values (not shown).

^a^Surgery duration differed significantly by implant type (*p* < .001). In post hoc testing, the Sigma (*p* = .014) and AGC (*p* = .001) implants had significantly shorter surgery durations than the other three implants. One patient with a NexGen implant had a surgery duration of 210 minutes; a sensitivity analysis excluding this patient yielded similar omnibus results (*p* = .004), and the remaining NexGen implants still had a mean surgery duration of 113.3 (SD 15.3) minutes.

^b^Tourniquet was used with the LCS more often than with the other implants (*p* = .009).

^c^On the night of the surgery, hemoglobin values did not differ by implant type. However, on postoperative Day 4, the more common implants (Sigma and AGC) were associated with higher hemoglobin values than the other implants (*p* = .020).
